# Supplementary material for: Genetic and biased agonist-mediated reductions in β-arrestin recruitment prolong cAMP signaling at glucagon family receptors
Source: J Biol Chem. 2020 Dec 4;296:100133. doi: 10.1074/jbc.RA120.016334 (PMC7948418; doi:10.1074/jbc.RA120.016334)
Supplement: Supplementary Figures and Tables [file mmc1.pdf]

Supporting Information

Genetic and biased agonist-mediated reductions in β-arrestin recruitment prolong cAMP signalling at glucagon family receptors

Ben Jones[1,#], Emma Rose McGlone[1], Zijian Fang[1,2], Phil Pickford[1], Ivan R Corrêa Jr[3], Atsuro Oishi[4,5], Ralf Jockers[4], Asuka Inoue[6], Sunil Kumar[7], Frederik Görlitz[7], Chris Dunsby[7], Paul MW French[7], Guy A Rutter[8,9], Tricia Tan[1], Alejandra Tomas[8,#], Stephen R Bloom[1].

[1]Section of Endocrinology and Investigative Medicine, Imperial College London, London, United Kingdom.
[2]Current address: Wellcome Trust – Medical Research Council Cambridge Stem Cell Institute and Department of Haematology, University of Cambridge, Cambridge, United Kingdom.
[3]New England Biolabs, Ipswich, USA.
[4]Université de Paris, Institut Cochin, INSERM, CNRS, F-75014 Paris, France.
[5]Current address: Department of Anatomy, Kyorin University Faculty of Medicine, Tokyo, Japan.
[6]Graduate School of Pharmaceutical Sciences, Tohoku University, Japan.
[7]Department of Physics, Imperial College London, London, United Kingdom.
[8]Section of Cell Biology and Functional Genomics, Imperial College London, London, United Kingdom.
[9]Lee Kong Chian School of Medicine, Nanyang Technological University, Singapore.

# Corresponding authors: Ben Jones (ben.jones@imperial.ac.uk) and Alejandra Tomas (a.tomas-catala@imperial.ac.uk)

Running title: Biased agonism at glucagon family receptors

Supplementary Table 1. Pharmacological characterisation of biased GLP-1R, GIPR and GCGR agonists. Mean parameter estimates ± SEM from responses depicted in Figure 3. * $p<0.05$, by one-way randomised block ANOVA with Dunnett's test *vs.* GLP-1, GIP and GCG, as appropriate. Note that for E_{max}, statistical comparison was performed prior to normalisation.

	**cAMP**					**βarr2**				
**GLP-1R**	**GLP-1**	**dHis1**	**Phe1**	**Gly2**	**dGln3**	**GLP-1**	**dHis1**	**Phe1**	**Gly2**	**dGln3**
E_{max} (% GLP-1)	100	101 ± 4	100 ± 4	95 ± 4	106 ± 5	100	66 ± 5 *	48 ± 6 *	93 ± 3	60 ± 4 *
Log EC_{50} (M)	-9.5 ± 0.2	-8.7 ± 0.2 *	-8.5 ± 0.2 *	-9.2 ± 0.2 *	-8.6 ± 0.2 *	-6.5 ± 0.2	-5.9 ± 0.1 *	-5.8 ± 0.0 *	-5.9 ± 0.1 *	-5.8 ± 0.0 *
Hill slope	1.7 ± 0.4	1.4 ± 0.3	1.4 ± 0.1	1.4 ± 0.1	1.7 ± 0.5	0.8 ± 0.1	2.0 ± 0.2 *	2.1 ± 0.2 *	1.0 ± 0.1	2.0 ± 0.3 *
Log (τ/KA)	9.5 ± 0.2	8.7 ± 0.1 *	8.5 ± 0.2 *	9.2 ± 0.2 *	8.6 ± 0.2 *	6.5 ± 0.1 *	5.6 ± 0.1 *	5.3 ± 0.1 *	5.9 ± 0.1 *	5.4 ± 0.1 *
**GIPR**	**GIP**	**dTyr1**	**Phe1**	**Gly2**	**dGln3**	**GIP**	**dTyr1**	**Phe1**	**Gly2**	**dGln3**
E_{max} (% GIP)	100	96 ± 6	99 ± 3	100 ± 4	110 ± 4	100	52 ± 6 *	65 ± 3 *	66 ± 7 *	23 ± 1 *
Log EC_{50} (M)	-10.0 ± 0.2	-8.5 ± 0.2 *	-8.7 ± 0.2 *	-9.1 ± 0.2 *	-7.6 ± 0.3 *	-7.1 ± 0.1	-6.3 ± 0.2 *	-6.0 ± 0.1 *	-6.5 ± 0.3	-6.5 ± 0.3
Hill slope	1.4 ± 0.1	1.3 ± 0.2	1.2 ± 0.1	1.6 ± 0.4	1.0 ± 0.1	0.6 ± 0.1	1.1 ± 0.1	1.2 ± 0.2	1.0 ± 0.2	85.7 ± 53.1
Log (τ/KA)	9.9 ± 0.2	8.5 ± 0.3 *	8.6 ± 0.2 *	9.1 ± 0.2 *	7.7 ± 0.3 *	7.2 ± 0.1	5.2 ± 0.2 *	5.5 ± 0.1 *	5.9 ± 0.1 *	4.0 ± 0.6 *
**GCGR**	**GCG**	**dHis1**	**Phe1**	**Gly2**	**dGln3**	**GCG**	**dHis1**	**Phe1**	**Gly2**	**dGln3**
E_{max} (% GCG)	100	108 ± 5	93 ± 2	99 ± 5	101 ± 5	100	85 ± 4 *	84 ± 4 *	43 ± 4 *	85 ± 4 *
Log EC_{50} (M)	-9.8 ± 0.2	-8.2 ± 0.2 *	8.9 ± 0.2 *	-8.1 ± 0.1 *	-8.3 ± 0.1 *	-7.4 ± 0.3	-5.9 ± 0.1 *	-6.4 ± 0.1 *	-5.8 ± 0.1 *	-5.8 ± 0.1 *
Hill slope	1.4 ± 0.1	1.4 ± 0.2	1.6 ± 0.1	1.7 ± 0.2	1.5 ± 0.1	0.6 ± 0.1	1.8 ± 0.2 *	1.7 ± 0.5 *	2.9 ± 0.4 *	1.8 ± 0.2 *
Log (τ/KA)	9.8 ± 0.2	8.3 ± 0.1 *	8.8 ± 0.2 *	8.1 ± 0.1 *	8.3 ± 0.1 *	7.6 ± 0.2	5.8 ± 0.1 *	6.5 ± 0.1 *	5.3 ± 0.1 *	5.8 ± 0.1 *

Supplementary Table 2. Responses to N-terminally substituted ligands in INS-1 832/3 and Huh7-GCGR cells. Mean parameter estimates ± SEM from insulin secretory responses depicted in Figure 4B, 4C and 4E, and 16-hour cAMP accumulation in Figure 4F. Note that the Hill slopes for GCG analogues in Huh7-GCGR cells are derived from the curves plotted from the pooled data, so no statistical comparisons are shown. * $p<0.05$, by one-way randomised block ANOVA with Dunnett's test *vs.* GLP-1, GIP and GCG, as appropriate. "n.c." indicates not calculated.

	**GLP-1 analogues - wild-type INS-1 832/3 cells**				
	**GLP-1**	**dHis1**	**Phe1**	**Gly2**	**dGln3**
E_{max}	4.0 ± 0.7	3.8 ± 0.8	4.9 ± 0.8 *	3.8 ± 0.6	5.0 ± 1.0 *
Log EC_{50} (M)	-8.5 ± 0.1	-8.6 ± 0.0	-7.4 ± 0.1 *	-9.2 ± 0.1 *	-7.5 ± 0.1 *
	**GIP analogues - wild-type INS-1 832/3 cells**				
	GIP	**dTyr1**	**Phe1**	**Gly2**	**dGln3**
E_{max}	4.2 ± 0.2	4.9 ± 0.3 *	5.1 ± 0.4 *	5.0 ± 0.3 *	5.4 ± 0.3 *
Log EC_{50} (M)	-8.3 ± 0.1	-7.4 ± 0.1 *	-6.9 ± 0.2 *	-7.8 ± 0.1 *	-6.4 ± 0.1 *
	**GCG analogues – wild-type INS-1 832/3 cells**				
	**GCG**	**dHis1**	**Phe1**	**Gly2**	**dGln3**
E_{max}	n.c	n.c	n.c	n.c	n.c
Log EC_{50} (M)	n.c	n.c	n.c	n.c	n.c
	**GCG analogues – GLP-1R KO INS-1 832/3 cells**				
	**GCG**	**dHis1**	**Phe1**	**Gly2**	**dGln3**
E_{max}	1.3 ± 0.1	1.4 ± 0.1	1.5 ± 0.2	1.6 ± 0.2	1.5 ± 0.2
Log EC_{50} (M)	-7.2 ± 0.5	-7.3 ± 0.3	-7.0 ± 0.8	-6.9 ± 0.4	-6.5 ± 0.5
	**GCG analogues – Huh7-GCGR cells**				
	**GCG**	**dHis1**	**Phe1**	**Gly2**	**dGln3**
E_{max}	46 ± 1	64 ± 2 *	64 ± 4 *	64 ± 4 *	67 ± 4 *
Log EC_{50} (M)	-8.8 ± 0.0	-8.6 ± 0.0 *	-9.0 ± 0.1 *	-8.4 ± 0.0 *	-9.1 ± 0.0 *
Hill slope	1.9	3.4	2.8	3.0	3.0

Supplementary Figure 1

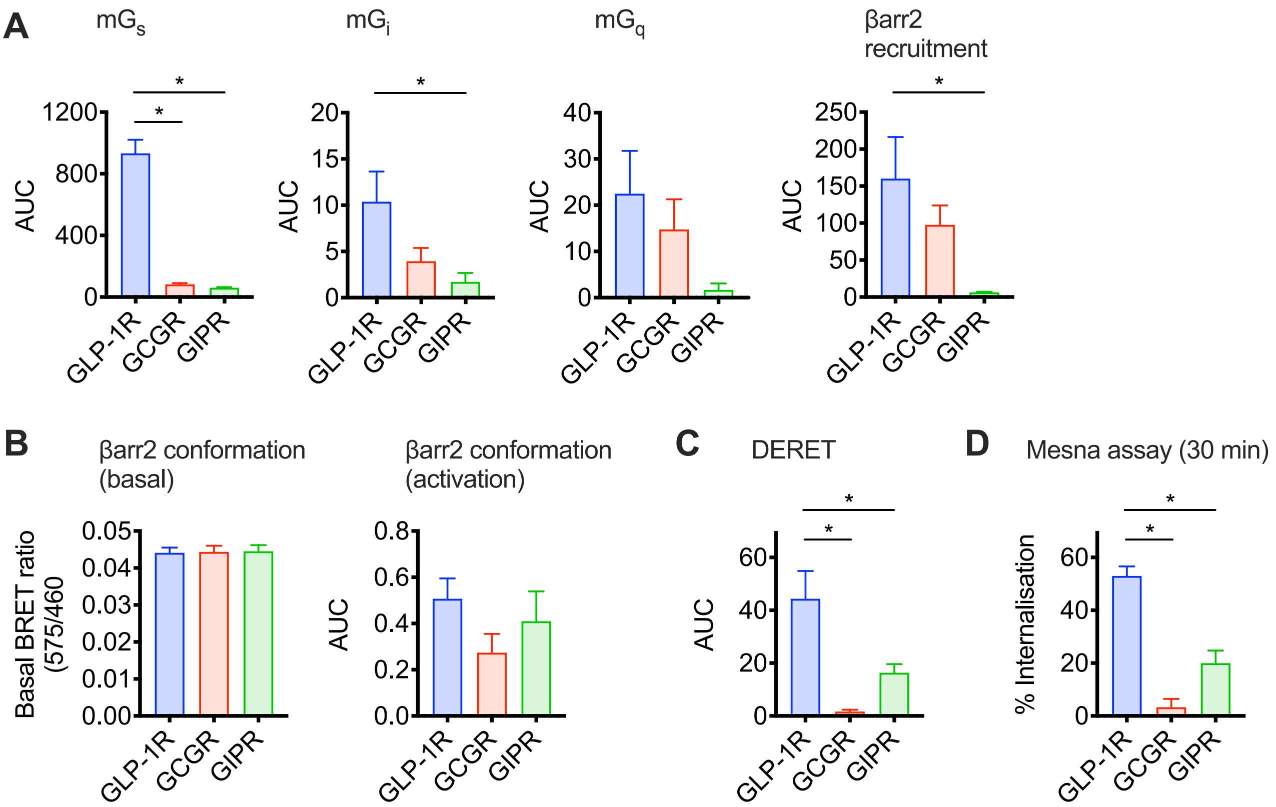

Supplementary Figure 1. Recruitment and signalling responses. (**A**) AUC analyses from nanoBiT kinetic responses shown in Figure 1A, with comparisons by one-way ANOVA with Tukey's test. (**B**) Basal (unstimulated) BRET ratio and AUC analysis from β-arrestin-2 activation BRET assay shown in Figure 1B, with comparisons by one-way ANOVA with Tukey's test. (**C**) AUC analysis from DERET assay shown in Figure 1C, with comparisons by randomised block one-way ANOVA with Tukey's test. (**D**) Statistical comparison of internalisation measured by reversible surface SNAP-labelling (see Figure 1D, E) using randomised block one-way ANOVA with Tukey's test. * $p<0.05$ by statistical test indicated in the text. Data are represented as mean ± SEM.

Supplementary Figure 2

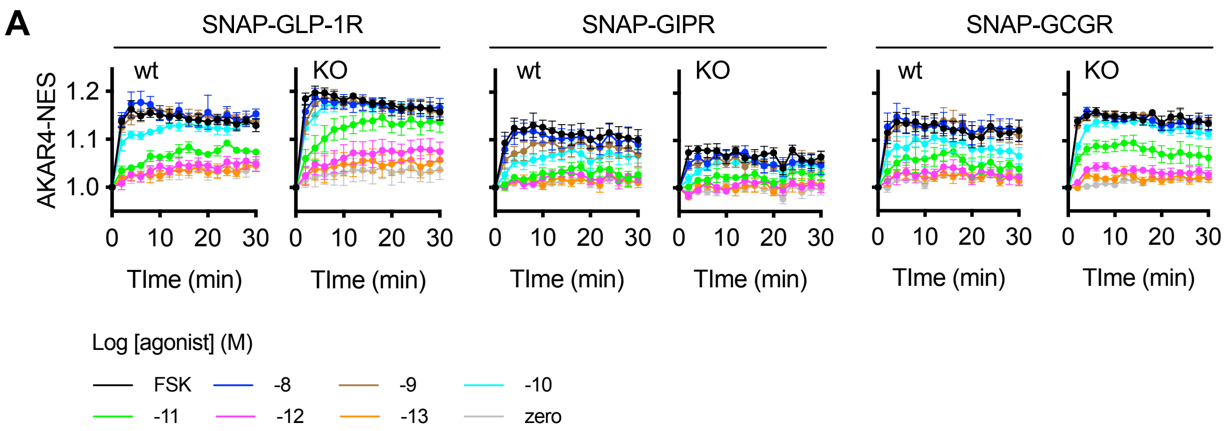

Supplementary Figure 2. Responses in wild-type *versus* dual β-arrestin knockout cells. (**A**) Cytoplasmic PKA activation in wild-type or dual β-arrestin knockout HEK293 cells transiently transfected with AKAR4-NES and indicated SNAP-tagged receptor, stimulated with indicated concentration of GLP-1, GIP or GCG, or forskolin (10 μM), with FRET signal indicated ratiometrically after normalisation to individual well baseline, n=5. Data are represented as mean ± SEM.
